# Examining *Escherichia coli* glycolytic pathways, catabolite repression, and metabolite channeling using Δ*pfk* mutants

**DOI:** 10.1186/s13068-016-0630-y

**Published:** 2016-10-10

**Authors:** Whitney D. Hollinshead, Sarah Rodriguez, Hector Garcia Martin, George Wang, Edward E. K. Baidoo, Kenneth L. Sale, Jay D. Keasling, Aindrila Mukhopadhyay, Yinjie J. Tang

**Affiliations:** 1 Department of Energy, Environmental and Chemical Engineering, Washington University, St. Louis, MO USA; 2 Sandia National Laboratory, Livermore, CA USA; 3 Joint BioEnergy Institute, Emeryville, CA USA; 4 Lawrence Berkeley National Laboratory, Biological Systems and Engineering Division, Berkeley, CA USA; 5 California Institute of Quantitative Biosciences (QB3), University of California, Berkeley, CA USA; 6 Department of Bioengineering, University of California, Berkeley, CA USA; 7 Department of Chemical and Biomolecular Engineering, University of California, Berkeley, CA USA; 8 Novo Nordisk Foundation Center for Biosustainability, Technical University of Denmark, Kogle Allé, DK2970 Hørsholm, Denmark

**Keywords:** ^13^C, Channeling, EMP, Metabolic modeling, Synthetic biology, Catabolite repression, Xylose

## Abstract

**Background:**

Glycolysis breakdowns glucose into essential building blocks and ATP/NAD(P)H for the cell, occupying a central role in its growth and bio-production. Among glycolytic pathways, the Entner Doudoroff pathway (EDP) is a more thermodynamically favorable pathway with fewer enzymatic steps than either the Embden–Meyerhof–Parnas pathway (EMPP) or the oxidative pentose phosphate pathway (OPPP). However, *Escherichia coli* do not use their native EDP for glucose metabolism.

**Results:**

Overexpression of *edd* and *eda* in *E. coli* to enhance EDP activity resulted in only a small shift in the flux directed through the EDP (~20 % of glycolysis flux). Disrupting the EMPP by phosphofructokinase I (*pfkA*) knockout increased flux through OPPP (~60 % of glycolysis flux) and the native EDP (~14 % of glycolysis flux), while overexpressing *edd* and *eda* in this Δ*pfkA* mutant directed ~70 % of glycolytic flux through the EDP. The downregulation of EMPP via the *pfkA* deletion significantly decreased the growth rate, while EDP overexpression in the Δ*pfkA* mutant failed to improve its growth rates due to metabolic burden. However, the reorganization of *E. coli* glycolytic strategies did reduce glucose catabolite repression. The Δ*pfkA* mutant in glucose medium was able to cometabolize acetate via the citric acid cycle and gluconeogenesis, while EDP overexpression in the Δ*pfkA* mutant repressed acetate flux toward gluconeogenesis. Moreover, ^13^C-pulse experiments in the Δ*pfkA* mutants showed unsequential labeling dynamics in glycolysis intermediates, possibly suggesting metabolite channeling (metabolites in glycolysis are pass from enzyme to enzyme without fully equilibrating within the cytosol medium).

**Conclusions:**

We engineered *E. coli* to redistribute its native glycolytic flux. The replacement of EMPP by EDP did not improve *E. coli* glucose utilization or biomass growth, but alleviated catabolite repression. More importantly, our results supported the hypothesis of channeling in the glycolytic pathways, a potentially overlooked mechanism for regulating glucose catabolism and coutilization of other substrates. The presence of channeling in native pathways, if proven true, would affect synthetic biology applications and metabolic modeling.

**Electronic supplementary material:**

The online version of this article (doi:10.1186/s13068-016-0630-y) contains supplementary material, which is available to authorized users.

## Background

**Fig. 1:**
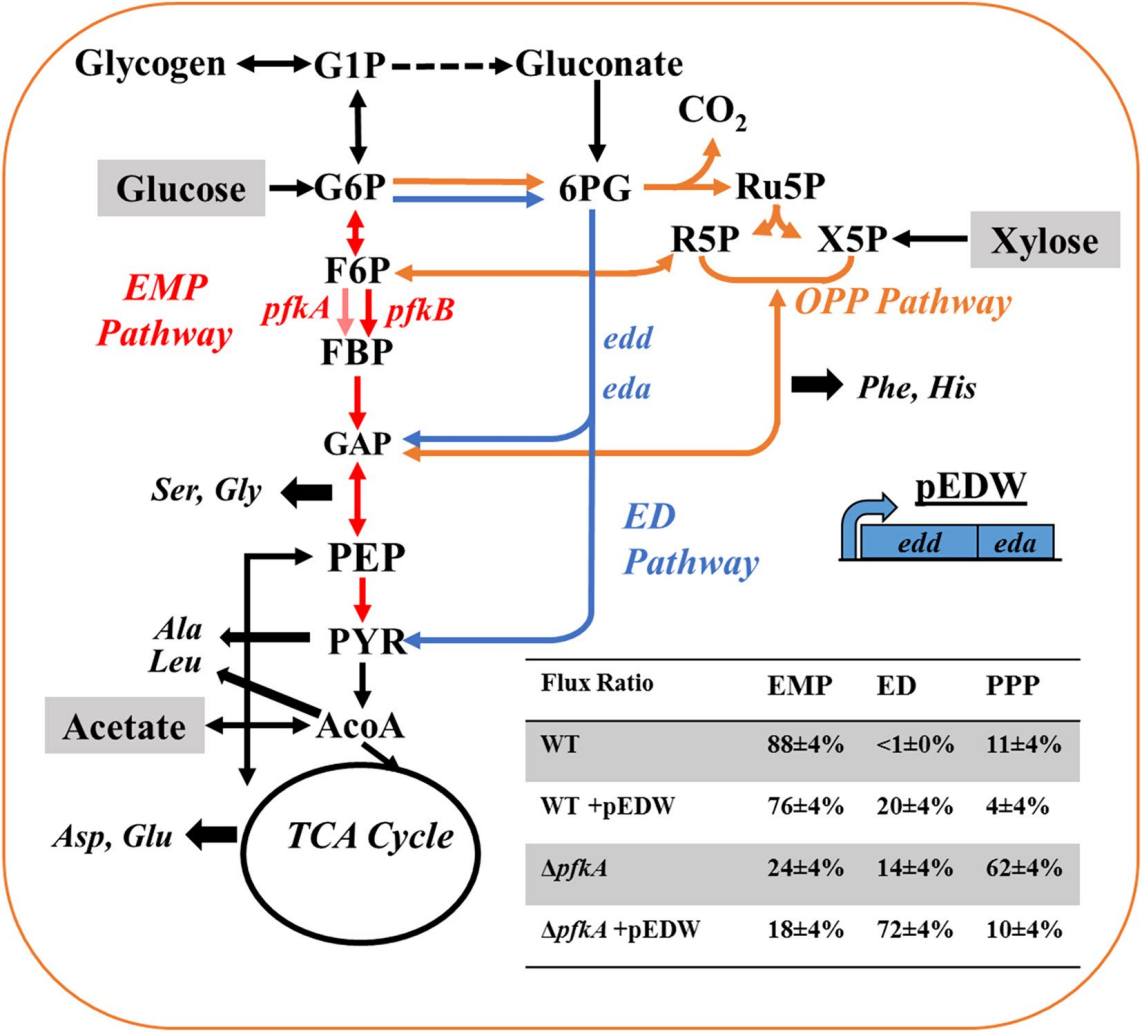
Redistribution of fluxes between the three primary glucose catabolic pathways: EMPP (*red*), EDP (*blue*), and OPPP (*orange*) via the knockout of *pfkA* and overexpression of EDP genes (*edd* and *eda*). Table on the *right* presents the estimated flux ratio between the three pathways for each strain. The *dashed arrows* represent a possible interference (unannotated) source from glycogen metabolism [27]

*Escherichia coli* have three native glycolytic pathways: EMPP, EDP, and OPPP. The EMPP employs ten enzymatic steps to yield two pyruvates, two ATP, and two NADH per glucose molecule [1], while OPPP serves as an oxidation route for NADPH synthesis. In *E. coli*, glucose metabolism mainly relies on the EMPP and the OPPP, while the EDP primarily remains inactive except during growth with gluconate [2]. The EDP utilizes only five enzymes to produce one pyruvate, one glyceraldehyde-3-phosphate, and one NADPH per glucose molecule (Fig. 1). With further conversion of glyceraldehyde-3-phosphate via the lower EMPP, the two pathways (EDP and EMPP) result in the same net production of pyruvate. However, the EMPP contains thermodynamic bottlenecks, comprising of fructose 1,6-bisphosphate aldolase and triose-phosphate isomerase. The EDP avoids both these unfavorable reactions (at the expense of ATP yield), and requires substantially less enzymatic protein than the EMPP [3]. Its end-products (glyceraldehyde 3-phosphate and pyruvate) are precursors of the non-mevalonate pathway, and thus, EDP upregulation has been proven to improve the yields of isoprenoids [4–6]. In addition, the EDP may also alleviate oxidative stress [7, 8] and improve NADPH generation without the loss of carbon (i.e., CO_2_) [9].

The EDP relies on two unique enzymes phosphogluconate dehydratase (*edd*) and 2-dehydro-3-deoxyphosphogluconate aldolase (*eda*), which are separate from the enzymes shared with the EMPP and OPPP. In wild-type (WT) *E. coli*, EDP flux is negligible. Δ*pgi* (G6P→F6P, encoding glucose-6-phosphate isomerase), Δ*pfk* (F6P→FBP, encoding phosphofructokinase), or overexpression of OPPP may redirect glycolytic fluxes and increase EDP activity [10, 11]. Δ*pgi* mutant has measureable EDP flux (up to ~15 % of glycolysis), but grew and consumed glucose more slowly than the WT strain. Δ*pgi* mutant may recover its growth rate by either increasing acetate overflow or activating the glyoxylate shunt after adaptive evolution [12]. Moreover, some bacterial species with exclusive EDP activity (e.g., *Rhodococcus opacus*) can coutilize glucose with other substrates [13]. Thus, we were curious if the presence of a highly active EDP could alleviate *E. coli* glucose catabolite repression and promote cell growth. To reorganize the glycolytic pathways, we engineered and characterized Δ*pfkA* mutants (deletion of phosphofructokinase I, important in the regulation of glycolysis-gluconeogenesis, Fig. 1). Through growth experiments and ^13^C-labeling, we elucidated *E. coli* physiological changes and its capability for simultaneous utilization of carbon substrates. Finally, Δ*pfkA* mutants also allowed us to evaluate the hypothesis of the existence of metabolite channeling in glycolytic pathways [14].

## Results

### Overexpression of EDP in *E. coli*

Overexpressing *edd* and *eda* in the wild-type (WT) strain reduced the flux (~20 %) through EMPP and OPPP. Despite the increased EDP activity, the growth rate of the EDP overexpressing strain decreased by ~30 % compared to WT (Table 1). To confirm the tradeoff from metabolic burdens imposed by antibiotics and plasmid/protein expression, we replaced EDP genes with the gene encoding for yellow fluorescent protein (YFP). The YFP-expressing strain had a similar reduced growth. A recent study found that the insertion of EDP (*pgi, zwf, pgl, edd, eda* from *Z. mobilis*) into *E. coli* chromosome also repressed the growth of the engineered strain [4].

**Table 1:** Strains/plasmids used

Plasmids/strains	Description	Maximal growth rate (h^−1^)	JBEI ICE codes	Source
Plasmids				
pBbE5c-YFP	Backbone vector expressing YFP	–	–	[48]
pEDW	Derived from pBbE5c-YFP with gene replaced by *edd* and *eda*	–	–	This study
Strains				
BW25113	Keio collection WT(*rrnB3 ∆lacZ4787 hsdR514 ∆araBAD567 ∆rhaBAD568 rph*-*1*)	0.78 ± 0.02	–	[15]
JW3887	BW25113 ∆*pfkA*	0.18 ± 0.02	–	
WH01	BW25113 (pBbE5c-YFP)	0.56 ± 0.07	JBEI-14575	This study
WH02	JW3887 (pBbE5c-YFP)	0.13 ± 0.01	JBEI-14585	This study
WH03	BW25113 (pEDW)	0.49 ± 0.02	JBEI-11465	This study
WH04	JW3887 (pEDW)	0.18 ± 0.01	JBEI-11468	This study

To further improve EDP flux, the Keio collection Δ*pfkA* (phosphofructokinase I, F6P→FBP) mutant was used [15]. The phosphofructokinase has two isoenzymes (*pfkA* and *pfkB*) with *pfkA* as the primary enzyme responsible for the conversion of F6P to FBP [16]. ^13^C-fingerprinting revealed that the Δ*pfkA* mutant (JW3887) distributed glucose flux through the OPPP (~62 %), the EDP (~14 %) and the EMPP (~24 %) (Fig. 1). The *pfkA* knockout increased cell lag phase, and reduced both cell growth rate and acetate overflow (Additional file 1: Figure S1). This is because the glycolytic flux reorganization can cause metabolic burdens, cofactor imbalances, and reduced carbon yield due to CO_2_ loss from the high OPPP flux [17]. Overexpressing EDP in the Δ*pfkA* mutant (resulting in strain WH04) raised the EDP flux to ~72 % and reduced the EMPP flux to ~18 % (Fig. 1). WH04 grew much faster than WH02 (the ∆*pfkA* mutant with YFP overexpression) (Table 1), demonstrating a beneficial impact of EDP in the absence of EMPP.

### Removal of glucose, carbon catabolite repression

**Fig. 2:**
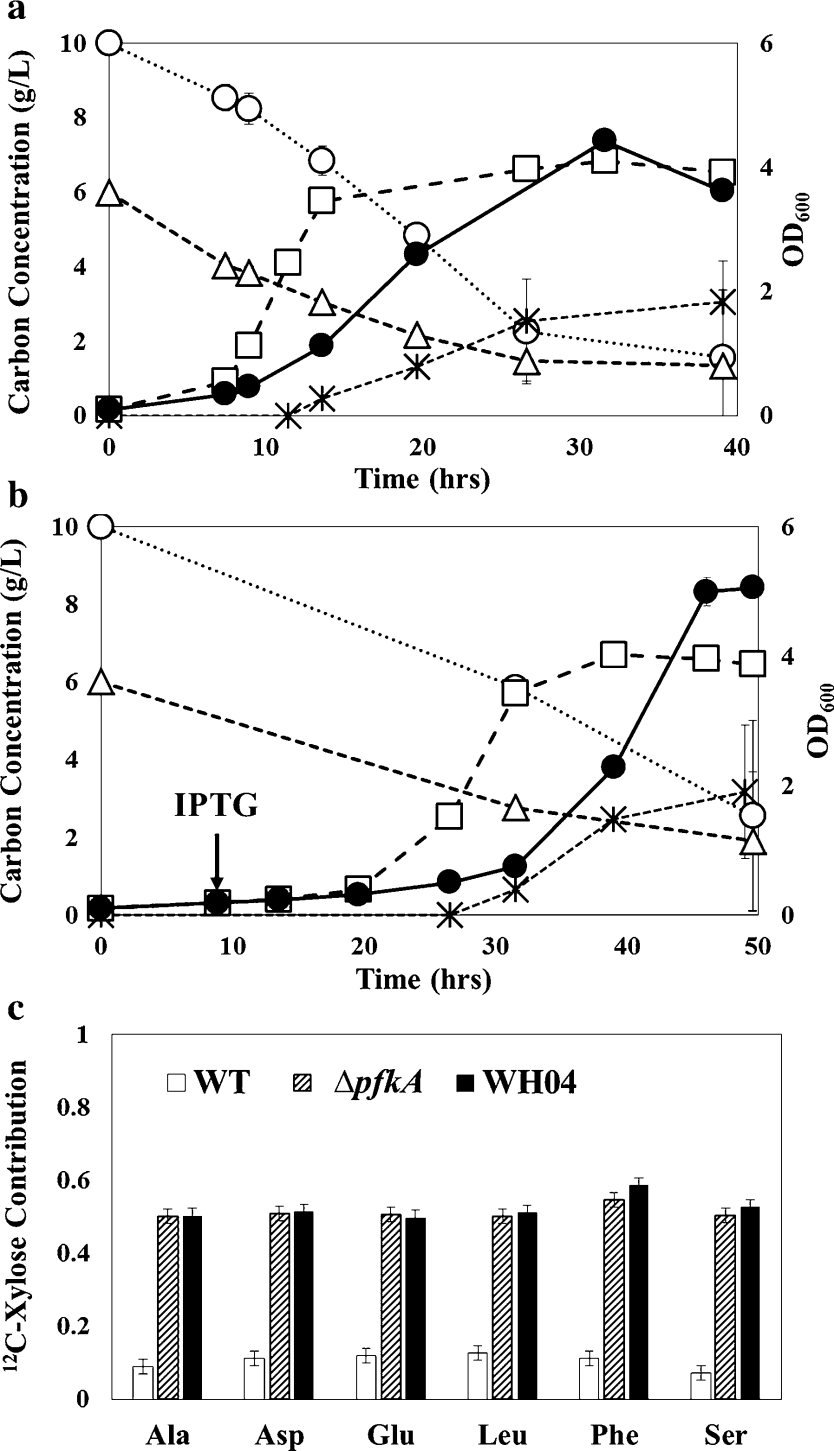
Glucose and xylose coutilization (n = 2). Profile of growth (*open square*), acetate production (*star*), and consumption of glucose (*open circle*) and xylose (*open triangle*) in mutant strains: **a** ∆*pfkA* mutant (JW3887) and **b** WH04 grown with glucose and xylose. OD_600 _of cultures grown solely with glucose are also shown (*closed circles*). **c** ^13^C isotopic labeling tracing in cultivations fed 10 g/L of ^13^C_6_-glucose and 6 g/L of unlabeled xylose. Xylose contribution (fraction of ^12^C) in proteinogenic amino acids is graphed for the three strains (2 % isotopic measurement errors were applied)

Lignocellulose hydrolysate contains glucose, xylose, and acetate. However, carbon catabolite repression (CCR) inhibits hosts from consuming diverse substrates simultaneously (i.e., *E. coli* mainly consumes glucose first in a glucose/xylose medium). The CCR is often explained by glucose inhibition on the synthesis of enzymes involved in catabolism of other carbon sources, while recent studies suggest that the presence of subpopulations within *E. coli* cultures can have different carbon utilization hierarchies [18, 19]. To investigate glucose catabolite repression, we grew the strains with a mixed carbon source of 10 g/L of glucose and 6 g/L of xylose, and measured the consumption of the two sugars (Fig. 2a, b). The Δ*pfkA* mutant (JW3887) simultaneously consumed glucose and xylose, and xylose coutilization nearly doubled the growth rate compared to its glucose-only culture (Fig. 2a). WH04 can also uptake xylose with glucose, and xylose addition reduced the strain’s lag phase (Fig. 2b). Moreover, the assimilation of xylose into biomass was determined through ^13^C-experiments (cultures fed with ^13^C_6_-glucose and unlabeled xylose). The fraction of xylose incorporated into proteinogenic amino acids during the exponential phase was measured (Fig. 2c). For both JW3887 and WH04, the xylose incorporation was ~50 % (Fig. 2c), much higher utilization than in the WT strain. Therefore, the removal of one pivotal EMP enzyme, phosphofructokinase I, alleviated glucose catabolite repression.

**Fig. 3:**
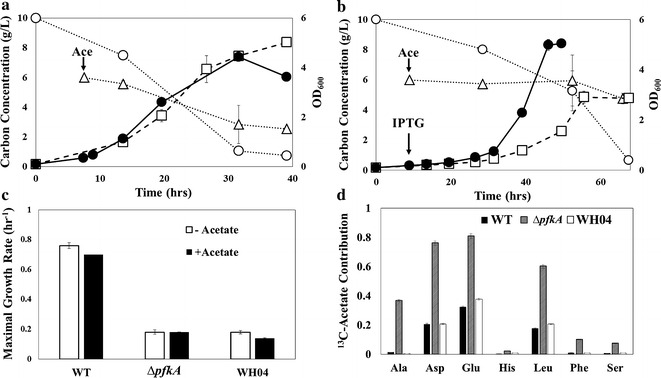
Glucose and acetate coutilization (n = 2). The growth (*open square*) and substrate consumption (glucose, *open circle*; acetate, *open triangle*) in mutant strains initially fed 10 g/L of glucose then supplemented with 6 g/L of sodium acetate (*denoted in figure*): **a** ∆*pfkA* (JW3887) and **b** WH04. OD_600_ of cultures grown solely with 10 g/L of glucose are also included (*closed circles*). **c** Maximal growth rate of WT, JW3887, and WH04 in cultures supplemented with 6 g/L of sodium acetate during early exponential phase (*black bar*). Control cultivations (no acetate; *white bar*) were supplemented with 6 g/L of sodium chloride to normalize for ion effects. **d** ^13^C tracing in cultivations fed 10 g/L of unlabeled glucose and 6 g/L of fully labeled ^13^C_2_-acetate. Acetate contribution (^13^C fraction) in proteinogenic amino acids are shown for the three strains

Acetate, another common component in lignocellulosic hydrolysate, has to be metabolized through gluconeogenesis [20]. Δ*pfkA* is expected to alleviate the EMPP repression on gluconeogenesis, and improve the coutilization of glucose and acetate. To test this hypothesis, the mutants (JW3887 and WH04) were grown with 10 g/L of glucose and supplemented with 6 g/L of sodium acetate during the early exponential phase (Fig. 3a–c). Although acetate may interfere with intracellular pH and hinder *E. coli* growth [21, 22], JW3887 was able to coutilize acetate with little effect on its growth. Acetate incorporation into biomass measured via cultures with unlabeled glucose and fully labeled acetate revealed that the mutant utilized acetate for biomass synthesis with enrichment of ^13^C into TCA and glycolysis-derived proteinogenic amino acids (Fig. 3d). In contrast, the glycolytic amino acids: Ala, Phe, and Ser, were unlabeled in WT under similar mixed carbon cultures, these amino acids being derived from PYR, PEP, and 3PG, respectively. Therefore, Δ*pfkA* (JW3887) could coutilize acetate to generate TCA cycle metabolites and glycolytic intermediates via upregulation of gluconeogenesis. However, introduction of EDP into the Δ*pfkA* mutant inhibited gluconeogenesis, as revealed with a lack of ^13^C Ala/Phe/Ser in WH04 biomass (Fig. 3d).

### Dynamic labeling pattern for sugar phosphates in Δ*pfkA* mutants

**Fig. 4:**
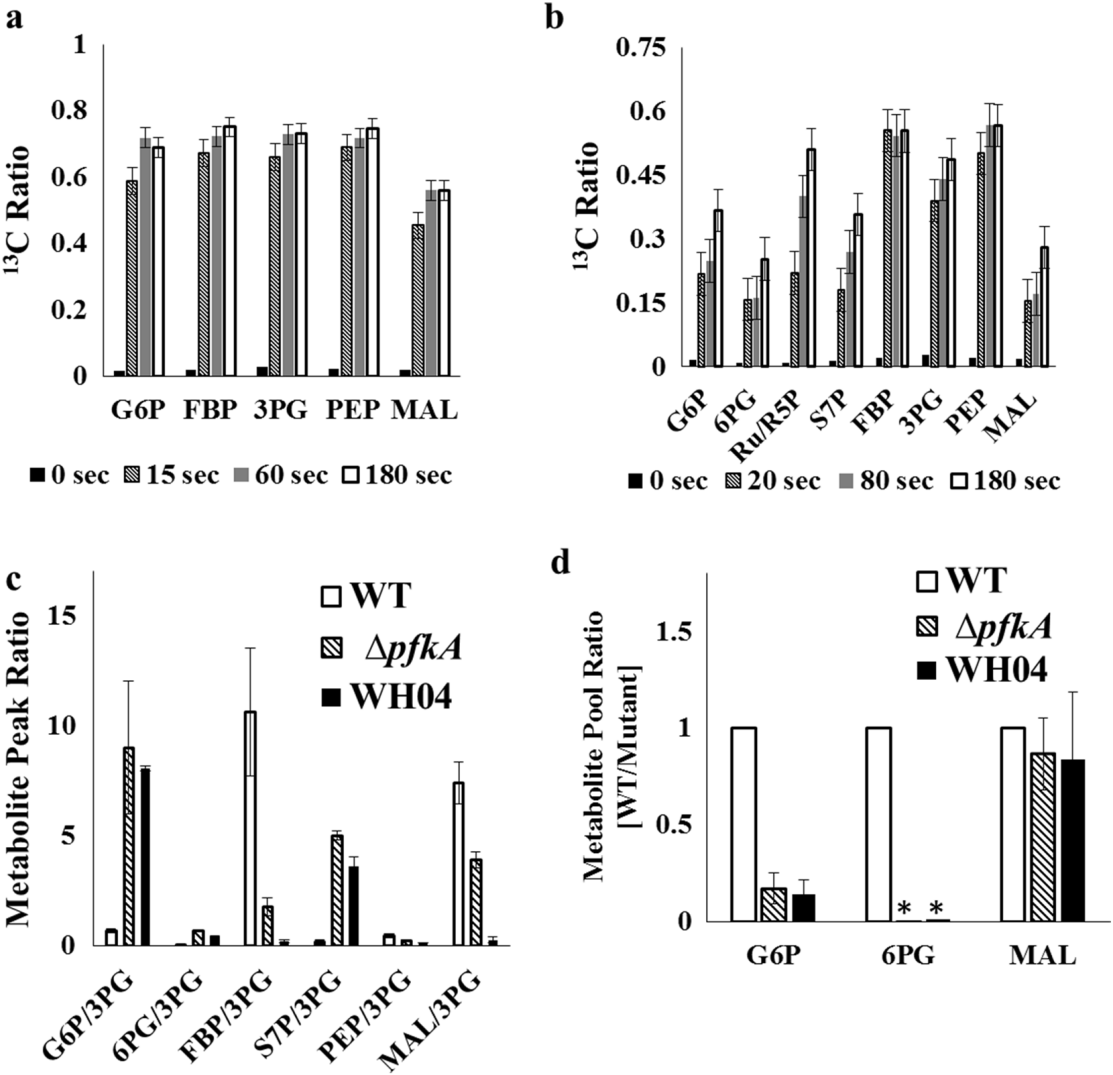
Dynamics labeling of central metabolites. Labeling dynamics in selected metabolites postpulse of ^13^C_6_-glucose into **a** Wild Type and **b** WH04. **c** Relative metabolite peak ratio of central metabolites in WT and ED mutant strains. The glycolysis key node (3PG) is used as the base of comparison. **d** Ratio of WT pool size to mutant pool size for G6P, 6PG, and MAL. The metabolite pools in two mutants, ∆*pfkA* (JW3887) and WH04, were measured via isotopomer ratio analysis. *Asterisk* 6PG measurement for WT was below detection

We grew WT and the Δ*pfkA* mutants (WH04 and JW3887) with unlabeled glucose into the mid-exponential growth phase, and then, we added uniformly ^13^C_6_-labeled glucose into the cultures. The resulting kinetics of ^13^C-labeling incorporation in key metabolites was examined. The WT demonstrated very fast metabolite turnover, such that most metabolites’ labeling reached isotopic steady state in 15 s (Fig. 4a and Additional file 2: Fig. S2). This observation is consistent with the rapid EMPP turnover rates previously reported [23]. In contrast, WH04 had lower rates of labeling incorporation. Interestingly, G6P and 6PG appeared to be labeled slower than their downstream metabolites (3PG and PEP) (Fig. 4b, Additional file 3: Fig. S3). Similarly, the ^13^C-pulse experiment for JW3887 also revealed that 3PG and R5P reached high isotopic ratios more quickly than their higher pathway metabolites (G6P and 6PG), revealing unusual labeling patterns in glycolytic intermediates (Additional file 4: Fig. S4).

**Fig. 5:**
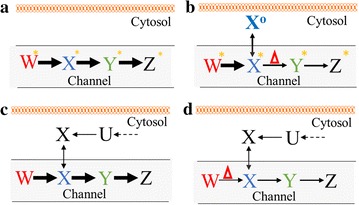
Schemes of hypothesized channeling and its influence on catabolite repression. An enzymatic pathway of sequential metabolites W, X, Y, and Z forms a channel. *Delta* represents the presence of a bottleneck that limits flux through the channel. **a** In a fully channeled network, most substrates exist within the channel with few substrates existing in cytosol. The pulse of ^13^C (*asterisk*) into the channeled pathway results in sequential and fast labeling of metabolites W, X, Y, and Z. **b** In a fractured channel, a disturbance in an enzyme (X→Y) causes accumulation of X in the cytosol, which is metabolically less active. After the pulse of ^13^C (*asterisk*), the labeling of bulk X* will be diluted by unlabeled X^o^ in the cytosol during LC–MS analysis. The downstream metabolites Y and Z may show faster ^13^C-labeling if their unlabeled pools in cytosol are small. In this case, the pulse of ^13^C and measurement of metabolite labeling dynamics can capture the features of channeling. **c** Substrate channeling can be another factor leading to CCR. The presence of channeling favors fast W conversion through the channeled pathway, while diffusion of cytosol metabolite (X) from secondary substrate (U) into channeled pathway becomes limited. **d** Catabolite repression is reduced if the channeled pathway flux is downregulated, allowing for cytosol X to diffuse into the channeled pathway

The unsequential ^13^C labeling patterns for glycolysis intermediates in Δ*pfkA* mutants could be explained by metabolite channeling (Fig. 5a): intermediates in metabolic pathways are passed from enzyme to enzyme without equilibration within the cellular medium [24]. For example, a large unlabeled metabolite pool formed outside of the ‘channel’ could dilute the labeled metabolite concentration, resulting in a slow labeling incorporation of the bulk metabolite, as measured by LC–MS methods. The downstream metabolites, however, retain their fast rates with their production primarily from the metabolite pool within the ‘channel’. In a previous study, in vivo evidence discovered that EMPP intermediates are mainly concentrated within a ‘channel’ with minimal mixing with the cytoplasmic pool in *E. coli* [14]. In vivo *studies in eukaryotes and* in vitro studies also suggest that EMPP and OPPP metabolites (e.g., 6PG) are highly channeled [25, 26]. In this study, disruption of EMPP channel by the knockout of *pfkA* caused a bottleneck in the conversion of F6P to FBP, which likely allowed hexose6P metabolites to accumulate within the cytosol. LC–MS peak abundances and isotopomer ratio analysis (Fig. 4c, d) further confirmed that ∆*pfkA* (JW3887) and WH04 had larger G6P and 6PG pool sizes than those in the WT. During ^13^C-pulse experiments, the unlabeled G6P and 6PG amassed in the cytosol could significantly slow the measured labeling of bulk hexose6P, causing ^13^C to appear more gradually than in their downstream metabolites (3PG and PEP). There could be other reasons for the observed labeling patterns in ∆*pfkA* mutants, such as activation of unknown pathways associated with glycolysis (e.g., Δ*edd* surprisingly increases glycogen accumulation [27]), experimental artifacts during ^13^C-metabolite sampling/extraction or heterogenity due to different cell sub-populations within the mutant cultures. However, channeling is a logical explanation given the labeling pattern difference between the WT and Δ*pfk* mutants for only certain intermediates.

### Fluxome response to changes in glycolytic pathway utilization

**Fig. 6:**
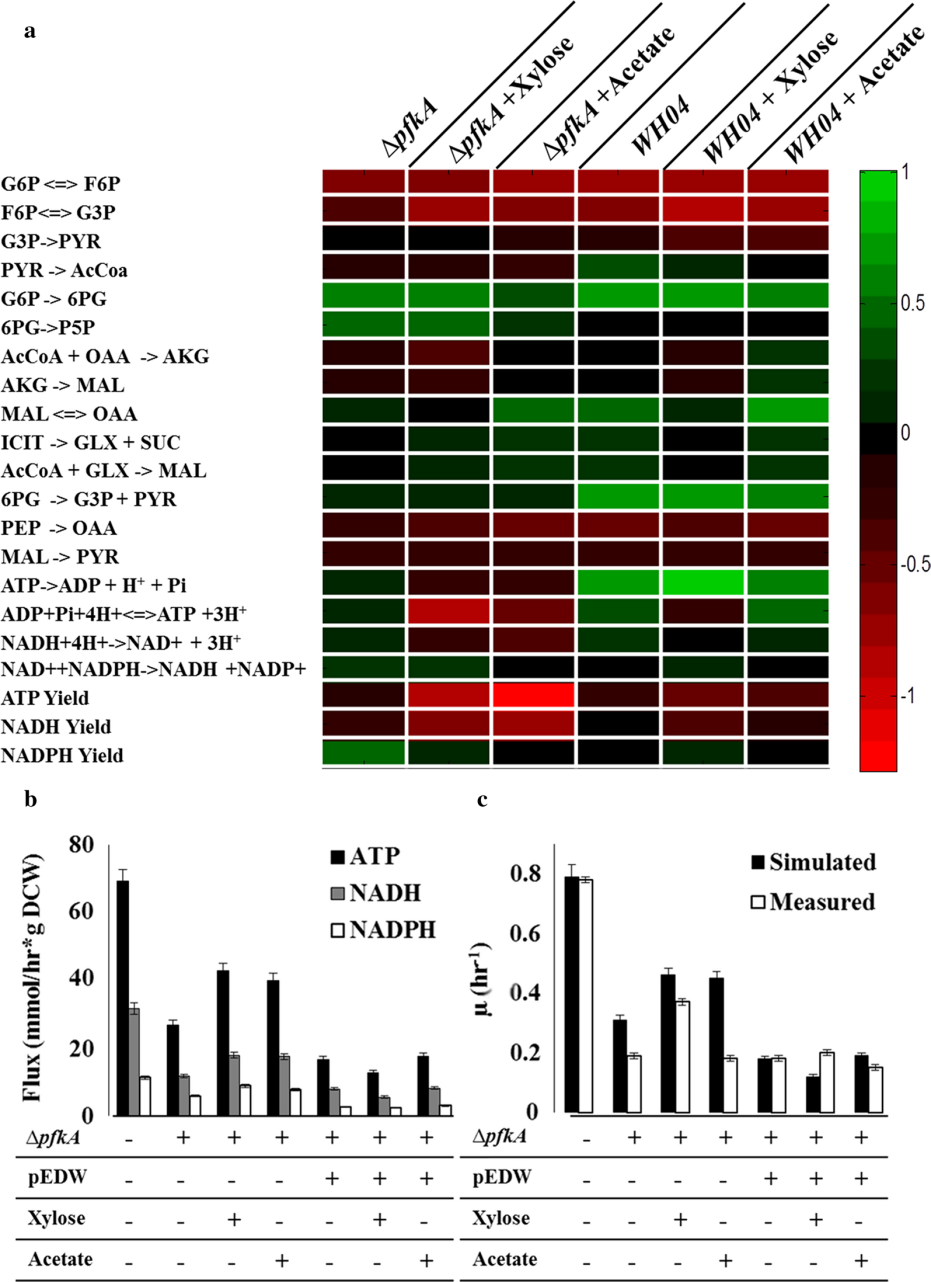
FBA of Δ*pfkA* (JW3887) and WH04. The FBA was constrained by the ^13^C-flux ratio and optimized for biomass accumulation. **a** Heat Map of the difference (Flux_mutant_-Flux_WT_) in the optimal fluxes (normalized to total carbon uptake rate, which was set to one) between mutant strains (Δ*pfkA*-JW3887 and WH04) and WT (*green* showed increased relative flux compared to wild type, *red* showed decreased relative flux compared to wild type). **b** Absolute total productions of ATP, NADH, and NADPH. **c** Simulated and measured growth rates of the different strains and growth conditions. The wild-type strain glucose uptake rate was assumed to be 8.5 mmol/h/g DCW

Flux balance analysis (FBA) was used to predict growth and flux distribution under different glycolytic pathways and carbon substrates [28]. Using the measured glycolytic flux ratios, substrate utilization, and acetate production as constraints, we performed FBA simulations on the mutant strains using maximal biomass growth as the objective function (Fig. 6a, b). FBA-predicted growth rate of WH04 agreed with the experimentally measured the glucose-based growth rate, while xylose and acetate co-utilization models show certain discrepanies. The measured growth rates of JW3887 were generally below the growth rates predicted by FBA (Fig. 6c). The knockout of *pfkA* may have unknown effects that cause a suboptimal metabolism for biomass synthesis. Unlike the WT strain, FBA predicted that Δ*pfkA* mutant and WH04 upregulated the glyoxylate shunt and maintained low activity of anaplerotic pathways for glucose metabolism (Fig. 6a). Compared to WT, the absolute fluxes of energy production (ATP, NADH, and NADPH) rate in mutants were reduced (Fig. 6b). FBA improved our overview of the central metabolic responses to our glycolysis flux reorganization.

## Discussion

Glycolytic pathways hold considerable control over carbon utilization and biosynthetic efficiency. The knowledge of their regulation is crucial for designing optimal microbial production strains. For example, the desire to use cheap cellulosic feedstock has prompted metabolic engineers to develop microbial hosts that utilize both 6- and 5-carbon sugars, simultaneously [29, 30]. However, carbon catabolite repression (CCR) prevents the simultaneous consumption of other carbon sources. CCR is believed to act in *E. coli* by preferentially directing resources to consume glucose more efficiently by regulating its sugar transporters and carbon degradation pathways [31]. Approaches such as adaptive evolution and overexpression of xylose transport and catabolic genes have been tested to minimize CCR [29]. The knockout of phosphoenolpyruvate-dependent glucose phosphotransferase system (PTS, the primary transport system for glucose) has also been shown to be an effective way to enable glucose and xylose coutilization [32–35], but has a negative effect on glucose uptake and growth rates. In this study, Δ*pfkA* mutants also showed no CCR and employed xylose co-consumption to increase its growth rate. Δ*pfkA* caused an increased pool size of G6P that may have inhibited PTS and decreased biomass accumulation [36, 37].

Gluconeogenesis is usually inactive under high glucose concentrations as it is regulated by phosphofructokinase and pyruvate kinase. The lack of EMPP flux could facilitate gluconeogenesis for utilization of non-sugar based substrates. This is seen in *R. opacus*, which lacks *pfk* activity and shows simultaneous gluconeogenesis and EDP for sugar and phenol co-utilization [13]. In this study, Δ*pfkA* mutants actively utilized acetate from the glucose medium. This observation is encouraging since acetate is a notorious inhibitor in *E. coli* cultivation, because acetate can freely diffuse through the cell membrane and disrupt the intracellular pH [21, 38, 39]. ^13^C-labeling analysis of Asp and Glu revealed that both JW3887 and WH04 actively employed the TCA cycle to degrade ^13^C-acetate in the presence of glucose. However, increased EDP flux in WH04 repressed the gluconeogenesis activity, since EDP overexpression drives high flux toward the lower portion of the EMPP.

Despite being more thermodynamically favorable and requiring ~5-fold less enzymatic protein [3], the *E. coli* mutants grew slower than WT after overexpression of EDP genes. One reason could be the lower ATP yield of EDP as compared to EMPP (this explanation is questionable since respiration remains the main ATP production route in all aerobic cultures via our FBA simulations). Another explanation is metabolite channeling. The intracellular environment contains high amounts of macromolecules (30–60 % of cell volume) creating a crowding effect within the cell that can reduce metabolite diffusion, and thus, channeling is an important mechanism for in vivo enzyme reactions [40]. Channeling can be naturally accomplished in the cell via electrostatic interactions, intramolecular tunnels, and small spatial distances between enzymes [41]. In eukaryotic cells, channeling is present for the TCA cycle and other pathways, as eukaryotes have organized their pathways within organelles [25, 26]. Pathways in *E. coli* have also been found to demonstrate forms of channeling, such as for fatty acid type II synthesis pathway [42]. As shown in this study and previous studies [43], *E. coli* EMPP channeling would significantly improve reaction thermodynamics and overcome kinetic obstacles. Thereby, it could be an important factor in WT and WH03 preference for EMPP rather than the EDP route.

The channeling process is difficult to study non-invasively, particularly in the central metabolism. In an early study by Shearer et al., *E. coli* was engineered so that the mutants could uptake both ^14^C-glucose and unlabeled sugar phosphates [14]. The authors found that the presence of unlabeled FBP in the culture medium did not dilute ^14^C EMPP intermediates or ^14^CO_2_, indicating a high degree of EMPP channeling. In this study, we performed ^13^C-pulse experiments to measure the labeling dynamics in WT and the Δ*pfkA* mutants (Additional files 2, 3, 4: Figure S2–S4). Our results supported the hypothesis that metabolites are not equilibrated in the cytoplasm (Fig. 5a, b). Previous ^13^C-pulse studies also examined the labeling dynamics for wild-type *E. coli* and *B. subtilis*, but the metabolites reached isotopic steady state within seconds of ^13^C-pulse [44, 45]. In this study, Δ*pfkA* mutants had slower metabolite transfer rates, and accumulated the upstream hexose6Ps outside of the channel (Fig. 5b). The resulting presence of two pools of the same metabolite allowed ^13^C-pulse experiments to detect unsequential metabolite labeling through glycolysis.

Channeling may have several implications on our understanding of cell physiology. First, metabolite channeling avoids diffusion limitations and thus significantly improves bioconversion efficiency [41]. Channeling would explain the robustness of natively evolved pathways for biosynthesis, encouraging synthetic biologists to replicate channeling as a strategy to improve heterologous pathway efficiency (e.g., colocalization/compartmentalization of overexpressed enzymes) [46]. Second, it necessitates that we rethink the in vivo Gibbs free energy or kinetics typically reported in studies, since the global metabolite pool measurement does not reflect the local substrate concentrations (The notion of ‘one perfectly mixed solution’ may be a poor reflection of the cell ‘in vivo’). This presents complications with metabolic modeling. Third, metabolite channeling could affect steady-state ^13^C-metabolic flux analysis, because channeling may prevent the carbon randomization during conversion of a symmetric metabolite or introduce bypass routes for ^13^C labeling [24]. Finally, we hypothesize that channeling could be another factor behind CCR. At high growth rates, the EMPP is capable of reaching fast glucose catabolic rates, such that metabolites derived from other substrates (e.g., xylose) cannot sufficiently diffuse into the EMPP channel (i.e., glucose catabolite repression). During slow growth conditions, glucose catabolic flux decreases. This favors diffusion of metabolites from cytosol into the EMPP channel and encourage the coutilization of the secondary substrate (Fig. 5c, d). In reality, inhibition of EMPP flux (e.g., removal of glucose transporter Δ*ptsG* or glucose-limiting chemostat cultures) allows *E. coli* to cometabolize glycerol with glucose [47].

## Conclusion

We have rewired the *E. coli* central metabolic network through a *pfkA* knockout and EDP overexpression. The altered strains alleviated glucose catabolite repression, and could be beneficial in biosynthesis from renewable cellulosic hydrolysates. Physiological analyses of different glycolytic strategy clearly revealed that despite the theoretical prediction that EDP could be a preferred glycolysis pathway based on both thermodynamics and the cost of enzymatic protein, the cell prefers EMPP for optimal growth. The results of ^13^C-pulse experiment revealed a form of glycolysis channeling that, if proven, could significantly affect our understanding of reaction thermodynamics, flux analysis, and synthetic biology applications.

## Methods

### Chemicals

Glucose, [1-^13^C] glucose, [U-^13^C] glucose, [U-^13^C] acetate, and all other chemicals unless otherwise stated were purchased from Sigma Aldrich (St. Louis, MO). Q5 High Fidelity 2X Master Mix was obtained from New England Biolabs (Ipswich, MA), and all other enzymes were purchased through Thermo Scientific (Waltham, MA).

### Construction of strains and plasmids

All plasmids were developed from a BglBrick expression vector, pBbE5c-YFP, which contains a ColE1 origin of replication, chloramphenicol resistance and LacUV5 promoter [48]. The genes, phosphogluconate dehydratase (*edd*) and 2-dehydro-3-deoxyphosphogluconate aldolase (*eda*) were PCR-amplified from genomic DNA of *E. coli* K-12 MG1655. The plasmid pEDW was assembled from the PCR-amplified genomic region from *edd* to *eda* into pBbE5c backbone via Gibson assembly. Primers used for plasmid construction are the following:

**eddeda01_fwd:** CTTTTAAGAAGGAGATATACATATGAATCCACAATTGTTACGCG.

**eddeda01_rev:** CGAGTTTGGATCCTTACAGCTTAGCGCCTTCTACAGC.

**vecbac01_fwd:** GGCGCTAAGCTGTAAGGATCCAAACTCGAGTAAGGATCTCC.

**vecbac01_rev:** CGCGTAACAATTGTGGATTCATATGTATATCTCCTTCTTAAAAGATCTTTTGAATTC.

Table 1 lists the resulting mutant strains, their JBEI ICE registry numbers, and growth rates. All strains generated at JBEI are available thought the JBEI Registry (https://acs-registry.jbei.org; [49]). The plasmid was first transformed into DH10B for plasmid propagation, and then verified by sequencing completed by Quintara (San Francisco, CA). After verification for no mutations, the plasmid was transformed into Keio collection strains, BW25113 (WT) and the ∆*pfkA* mutant (JW3887) [15], obtained from the Yale *E. coli* Genetic Stock Center (New Haven, CT).

### Media composition and growth conditions

Seed cultures were grown in LB for ~12 h, then inoculated (2 % v/v) in M9 minimal medium overnight. Strains were then inoculated in fresh M9 minimal medium (~2 % v/v). The M9 medium (pH = 7–7.5) consisted of 11 g/L of 5× M9 salts (Sigma Aldrich, St. Louis, MO), 2 mM MgSO_4_·7H_2_O, 0.1 mM CaCl_2_·2H_2_O, 1 mg/L of Thiamine HCl, 3 mg/L of FeSO_4_·7H_2_O, and trace minerals. Initial sugar concentrations were 10 g/L of glucose and 6 g/L of xylose. For mutant cultures, 30 mg/L chloramphenicol and 10 mg/L kanamycin were supplied as needed. 50 µM Isopropyl *β*-d-1-thiogalactopyranoside (IPTG) was used to induce plasmid borne genes at early exponential growth phase. To study acetate coutilization, 6 g/L sodium acetate (or 6 g/L NaCl served as a control) was added at early phase of mutant cultures (OD_600_ ~ 0.2, pH ~ 7.5) with the IPTG induction. All cultivations were aerobic and conducted in 5 mL cultures in test tubes on a rotary shaker at 250 rpm at 37 °C. For labeling experiments, unlabeled glucose was replaced with 100 % [1-^13^C] glucose or 100 % [U-^13^C] glucose, and unlabeled acetate was replaced with [U-^13^C] acetate.

## ^13^C-pulse experiments

Strains were grown in approximately 70 mL cultures in 250-mL Erlenmeyer flasks on a rotary shaker at 250 rpm with 2 g/L unlabeled glucose M9 media. In all ^13^C-pulse experiments, cultures in exponential growth phase (pH = 6–7) were pulsed with 5 mL–60 g/L ^13^C_6_-glucose stock solution. To measure the ^13^C-incorporation into glycolysis metabolites over time, cultures were harvested at different time points (from 15 s to 3 min). The harvested samples were quenched using the procedure of Fast-Cooling [50]. Specifically, ~10 mL culture was poured into a 50-mL falcon tube containing 2 mL ice-cold M9 medium (no carbon source) and the tube was immediately immersed in liquid N_2_. To facilitate heat transfer and avoid ice formation in the sample solution during the liquid N_2_ bath, the sample solutions were manually agitated using a digital thermometer. The sample solution could be cooled to 0 °C in 10 s. The ice-cold samples were centrifuged at 0 °C for 3 min, and the pellets were stored at −80 °C until metabolite extraction.

### Sugar measurement

Supernatant samples were taken in parallel experiments separate from cultures used for optical density measurement to reduce the loss of volume. At each time point, at least 150 µL of culture was extracted, centrifuged to remove cell biomass, and stored at −20 °C. Samples were diluted by 2× and then filtered through 0.22 μm centrifugal filters. Samples were run on an Agilent Technologies 1200 series HPLC equipped with an Aminex H column, and the concentrations were estimated based on standard curves [51].

### Amino acid extraction and GC–MS analysis

Amino acid extraction and GC–MS analysis were performed as described previously [52]. Briefly, cell pellets from 5 mL cultures taken during the exponential phase were washed with 0.9 % (w/v) of NaCl solution, and then hydrolyzed in 6 M HCl at 100 °C. The resulting amino acids were derivatized by *N*-*tert*-butyldimethylsilyl-*N*-methyltrifluoroacetamide prior to GC–MS analysis. For isotopic tracing, we used the fragments [M-15]^+^ or [M-57]^+^ (containing the entire amino acid backbone) and [M-159]^+^ or [M-85]^+^ (containing the amino acid backbone after loss of its first carbon). The natural isotopic abundance of derivatized amino acids was corrected using a reported algorithm [53]. The mass isotopomer distributions of alanine and serine for the strains used are given in Additional file 5: Table S1.

1-^13^C glucose was used to measure in vivo glucose catabolism: the OPPP cleaves the labeled carbon as CO_2_; the EDP produces the first carbon labeled pyruvate, while EMPP results in the labeled carbon present in the third position of pyruvate [1]. GC–MS analysis of alanine (synthesized from pyruvate) can reveal this positional labeling as further described previously [8, 13, 54]. Equations (1–3) were solved for estimation of the flux ratios between the three pathways (without considering metabolite channeling effect):

1 $$Ala_{100} = \frac{{v_{\text{EDP}} }}{{2v_{\text{EDP}} + 2v_{\text{EMPP}} + \frac{5}{3}v_{\text{OPPP}} }} ,$$

2 $$Ala_{000} = \frac{{\frac{5}{3}v_{\text{OPPP}} + v_{\text{EDP}} + v_{\text{EMPP}} }}{{2v_{\text{EDP}} + 2v_{\text{EMPP}} + \frac{5}{3}v_{\text{OPPP}} }} ,$$

3 $$Ala_{001} = \frac{{v_{\text{EMPP}} }}{{2v_{\text{EDP}} + 2v_{\text{EMPP}} + \frac{5}{3}v_{\text{OPPP}} }} ,$$

where *v*_EDP_, *v*_EMPP_, and *v*_OPPP_ are the estimated fluxes for the EDP, EMPP, and OPPP, respectively. *Ala*_xxx_ are the fractions of the isotopomer determined by fragmentation data, with 1 denoting a ^13^Carbon isotope. For example, *Ala*_100_ is the fraction of alanine labeled at carbon position 1, a unique isotopomer produced by the EDP. The calculated flux ratios are estimations, which assume the reactions are irreversible.

### Metabolite extraction and LC–MS/MS analysis

After quenching, the cell pellet was suspended in 1 mL of cooled 7:3 methanol:chloroform mixture. These samples were then placed on a rotary shaker at 250 rpm, overnight at 4 °C. The methanol layer was separated with the addition of 500 µL of water, then filtered through an Amicon Ultra centrifuge filter (3000 Da; EMD Millipore, Billerica, MA), lyophilized (−50 °C) and reconstituted in 100 µL of acetonitrile–water (6:4, v/v). The samples were then analyzed via liquid chromatography-mass spectrometry (LC–MS) using a SeQuant Zic-pHILIC column (EMD Millipore, Billerica, MA, USA) in an Agilent Technologies 1200 Series HPLC system. The mobile phase was composed of 15 mM ammonium carbonate (Sigma-Aldrich, St. Louis, MO, USA) in water (solvent A) and 15 mM ammonium carbonate in 75 % acetonitrile and 25 % water (solvent B). A flow rate of 0.2 mL/min was used, unless stated otherwise. Metabolites were separated via gradient elution under the following conditions: 100 % B (0 min), 82 % B (4.4 min), 72 % B (7.7 min), 60 % B (9.7 min), 100 % B (10.2 min), 100 % B (12.5 min), 100 % B (13 min, 0.4 mL/min), and 100 % B (18.5 min, 0.4 mL/min). The HPLC system was coupled to an Agilent Technologies 6210 series time-of-flight mass spectrometer (for LC–TOF MS) via a MassHunter workstation (Agilent Technologies, USA). A split ratio of 1:4 was used throughout. Drying and nebulizing gases were set to 10 L/min and 25 lb/in.^2^, respectively, and a drying-gas temperature of 300 °C was used throughout. Electrospray ionization (ESI) was conducted in the negative ion mode and a capillary voltage of 3500 V was utilized. The acquisition range was from 70 to 1000 *m*/*z*, and the acquisition rate was 0.86 spectra/s. Metabolite mass isotopomer distribution was determined based on the ratio of the integrated peak area of the chosen isotopomer to the sum of all the integrated peak areas of the possible isotopomers for the given metabolite.

### Estimation of pool size via isotope-ratio approach

The relative pool size of key metabolites (G6P, 6PG, and 3PG) was measured via an isotope-based ratio approach, as modified from [55]. Wild-type (BW25113) was cultivated in 5 g/L of ^13^C_6_-glucose and 1 g/L of fully labeled ^13^C-sodium bicarbonate, which generated ^13^C-metabolites as internal standards. The fully labeled WT biomass and the unlabeled mutant cultures were quenched using liquid N_2_ as described earlier, then mixed together at 1:1 volume ratio for metabolite extraction. The ratio of WT metabolite pool size (labeled peak area) to the mutant strains metabolite pool size (unlabeled peak area) was determined using LC–MS, which provided a qualitative comparison of metabolite pool sizes between WT and mutants.

### Flux balance analysis constrained by measured flux ratios

The *E. coli* Genome Scale Model iJO1366 (2251 metabolic reactions) was adapted for metabolic modeling of mutants under the different growth conditions [56]. Flux balance analysis (FBA) used the following objective function:

Maximize µ

Subject to: S · v = 0

$${\text{lb}} \le v \le {\text{ub}}$$

where S represents the stoichiometric matrix, *v* represents the matrix of reaction rates (fluxes), while matrices lb and ub are the lower and upper bounds, respectively. Glucose, xylose, and acetate uptake rates were fixed to measured average values with 5 % presumed variation. Flux ratios determined by ^13^C-analysis were used to constrain fluxes through EMPP, EDP, and OPPP (Fig. 1). As the activities of *pfkA* and *pfkB* were lumped in iJO1366, the PFK reaction was not constrained. All generated fluxes were normalized to total substrate uptake rate.

## Additional files

10.1186/s13068-016-0630-y Glucose consumption in (A) ∆*pfkA* (JW3887) and (B) WH04.

10.1186/s13068-016-0630-y Dynamics of ^13^C-labeling pulse experiment for WT (BW25113).

10.1186/s13068-016-0630-y Dynamics of ^13^C-labeling pulse experiment for WH04.

10.1186/s13068-016-0630-y Kinetics of ^13^C-isotopic incorporation in ∆*pfkA* (JW3887) culture taken immediately after ^13^C_6_-glucose pulse.

10.1186/s13068-016-0630-y Mass isotopomer distribution of alanine and serine.

## Abbreviations

3PG: 3-phosphoglycerate
6PG: 6-phosphogluconate
AcCoA: acetyl-CoA
F6P: fructose 6-phosphate
FBP: fructose 1,6-bisphosphate
G1P: glucose 1-phosphate
G6P: glucose 6-phosphate
GAP: glyceraldehyde phosphate
MAL: malate
PEP: phosphoenolpyruvate
PYR: pyruvate
Ru5P: ribulose 5-phosphate
R5P: ribose 5-phosphate
S7P: sedoheptulose 7-phosphate
X5P: xylulose 5-phosphate
