# Supplementary figures and images for: Examining Escherichia coli glycolytic pathways, catabolite repression, and metabolite channeling using Δpfk mutants
Source: Biotechnol Biofuels. 2016 Oct 10;9:212. doi: 10.1186/s13068-016-0630-y (PMC5057261; doi:10.1186/s13068-016-0630-y)

**Figure S1. Glucose Consumption in (A) ∆*pfkA* (JW3887) and (B) WH04** (both strains produce much less acetate than WT).


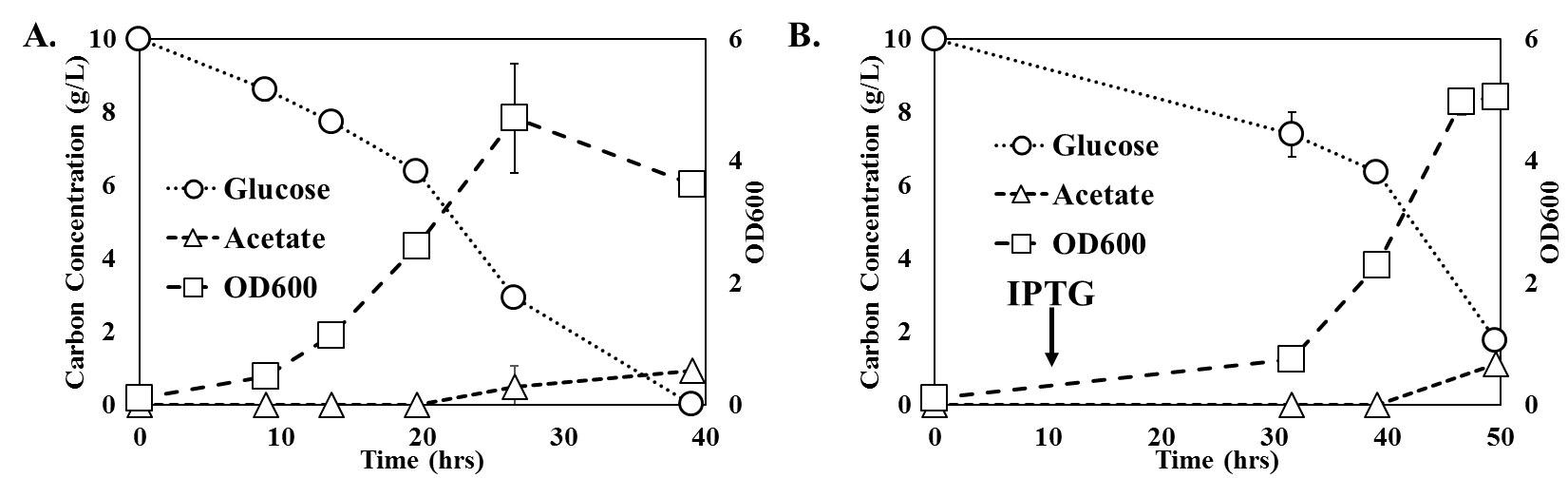

Supplement: Supplementary file 1 — 10.1186/s13068-016-0630-y Glucose consumption in (A) ∆pfkA (JW3887) and (B) WH04. [file 13068_2016_630_MOESM1_ESM.docx]

**Figure S2. Dynamics of 13C-labeling Pulse Experiment for WT (BW25113).**

**
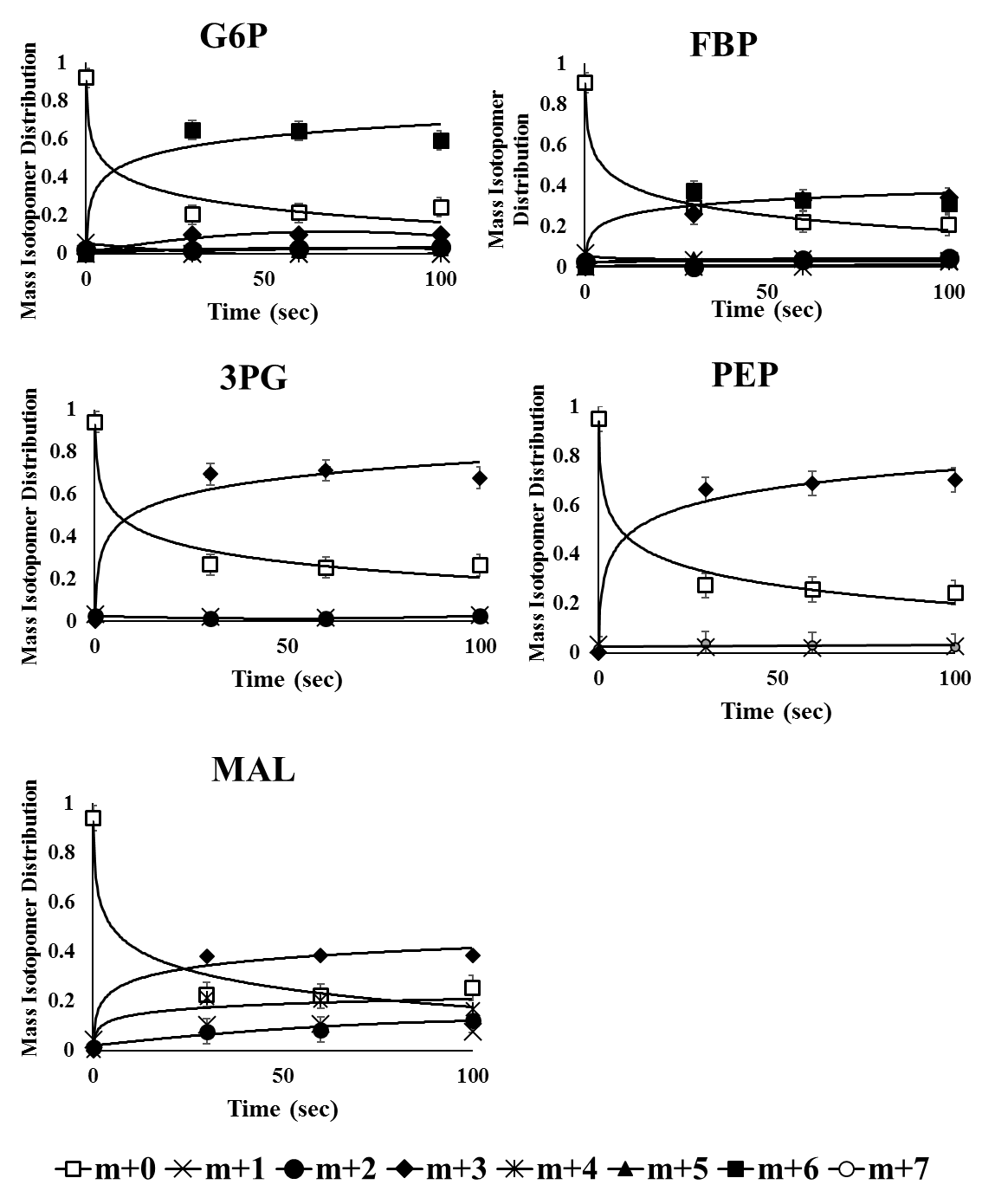
**

Supplement: Supplementary file 2 — 10.1186/s13068-016-0630-y Dynamics of 13C-labeling pulse experiment for WT (BW25113). [file 13068_2016_630_MOESM2_ESM.docx]

**Figure S3. Dynamics of 13C-labeling Pulse Experiment for WH04.**

**
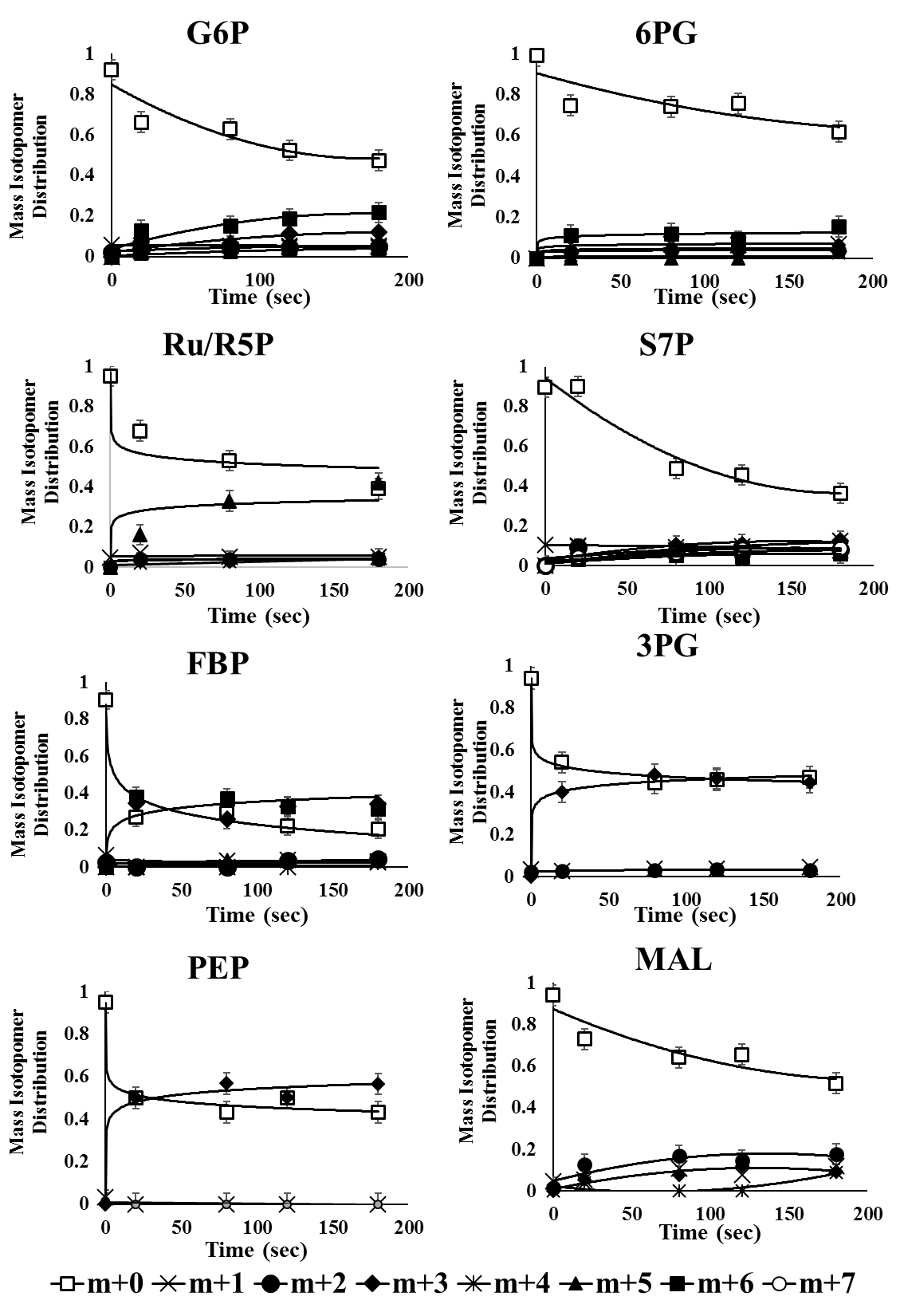
**

Supplement: Supplementary file 3 — 10.1186/s13068-016-0630-y Dynamics of 13C-labeling pulse experiment for WH04. [file 13068_2016_630_MOESM3_ESM.docx]
