# Supplementary material for: Examining Escherichia coli glycolytic pathways, catabolite repression, and metabolite channeling using Δpfk mutants
Source: Biotechnol Biofuels. 2016 Oct 10;9:212. doi: 10.1186/s13068-016-0630-y (PMC5057261; doi:10.1186/s13068-016-0630-y)
Supplement: Supplementary file 4 — 10.1186/s13068-016-0630-y Kinetics of 13C-isotopic incorporation in ∆pfkA (JW3887) culture taken immediately after 13C6-glucose pulse. [file 13068_2016_630_MOESM4_ESM.docx]

**Figure S4. ^13^C-Isotopic Incorporation kinetics in ∆*pfkA*(JW3887) glucose culture taken immediately after ^13^C_6_-Glucose pulse. The early time point labeling shows the the difference in labeling pattern that is difference from the WT.**

**
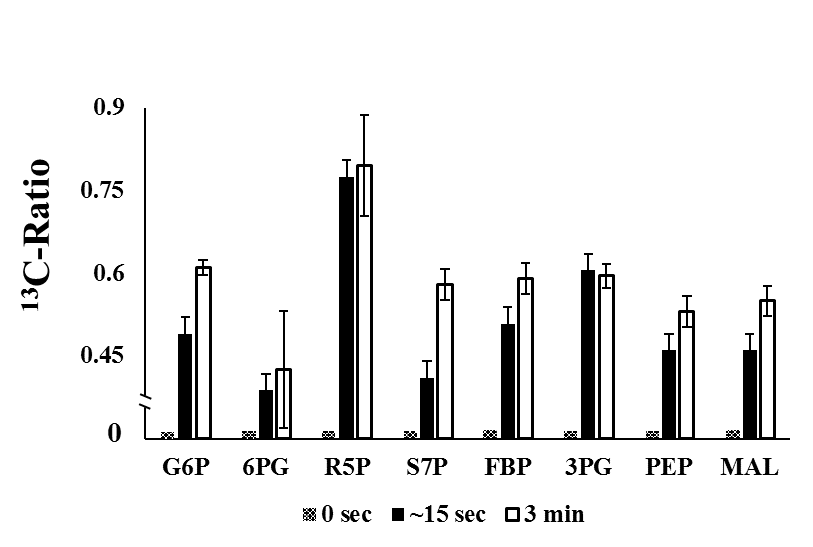
**
