# Supplementary material for: Examining Escherichia coli glycolytic pathways, catabolite repression, and metabolite channeling using Δpfk mutants
Source: Biotechnol Biofuels. 2016 Oct 10;9:212. doi: 10.1186/s13068-016-0630-y (PMC5057261; doi:10.1186/s13068-016-0630-y)
Supplement: Supplementary file 5 — 10.1186/s13068-016-0630-y Mass isotopomer distribution of alanine and serine. [file 13068_2016_630_MOESM5_ESM.docx]

**Table S1. Mass Isotopomer Distribution of Alanine and Serine**

|  | BW25113 | WH03 | JW3887 | WH04 |
| --- | --- | --- | --- | --- |
| [M-57]^+^ | **Alanine** | | | |
| m+0 | 0.53 | 0.51 | 0.67 | 0.54 |
| m+1 | 0.46 | 0.49 | 0.33 | 0.46 |
| m+2 | 0.01 | 0.00 | 0.00 | 0.00 |
| m+3 | 0.00 | 0.00 | 0.00 | 0.00 |
| [M-85]^+^ |  |  |  |  |
| m+0 | 0.54 | 0.62 | 0.74 | 0.95 |
| m+1 | 0.46 | 0.38 | 0.26 | 0.05 |
| m+2 | 0.00 | 0.00 | 0.00 | 0.00 |
| [M-57]^+^ | **Serine** | | | |
| m+0 | 0.56 | 0.59 | 0.71 | 0.91 |
| m+1 | 0.44 | 0.42 | 0.29 | 0.09 |
| m+2 | 0.00 | 0.00 | 0.00 | 0.00 |
| m+3 | 0.00 | 0.00 | 0.00 | 0.00 |
| [M-159]^+^ |  |  |  |  |
| m+0 | 0.56 | 0.60 | 0.72 | 0.94 |
| m+1 | 0.44 | 0.41 | 0.28 | 0.06 |
| m+2 | 0.00 | 0.0 | 0.00 | 0.00 |

Note: Instrumental measurement errors are below 0.02.
